# Influenza Virus Segment Composition Influences Viral Stability in the Environment

**DOI:** 10.3389/fmicb.2018.01496

**Published:** 2018-07-09

**Authors:** Thomas Labadie, Christophe Batéjat, Jean-Claude Manuguerra, India Leclercq

**Affiliations:** ^1^Institut Pasteur, Department of Infection and Epidemiology, Environment and Infectious Risks Unit, Laboratory for Urgent Response to Biological Threats (CIBU), Paris, France; ^2^Sorbonne Paris Cité (Cellule Pasteur), Paris Diderot University, Paris, France

**Keywords:** influenza A virus, environment, persistence, neuraminidase, hemagglutinin, reassortant viruses, waterborne pathogens, codon usage bias

## Abstract

The transmission routes of Influenza A viruses (IAVs) submit virus particles to a wide range of environmental conditions that affect their transmission. In water, temperature, salinity, and pH are important factors modulating viral persistence in a strain-dependent manner, and the viral factors driving IAV persistence remain to be described. We used an innovative method based on a real-time cell system analysis to quantify viral decay in an environmental model. Thus, we identified the viral hemagglutinin (HA) and neuraminidase (NA) as the main proteins driving the environmental persistence by comparing the inactivation slopes of several reassortant viruses. We also introduced synonymous and non-synonymous mutations in the HA or in the NA that modulated IAV persistence. Our results demonstrate that HA stability and expression level, as well as calcium-binding sites of the NA protein, are molecular determinants of viral persistence. Finally, IAV particles could not trigger membrane fusion after environmental exposure, stressing the importance of the HA and the NA for environmental persistence.

## Introduction

Influenza A viruses (IAVs) have a wide host range, which allows them to spread almost everywhere on the planet. In nature, the H1N1 subtype infects several hosts such as domestic and aquatic birds, humans, swine, or dogs and thus spreads through a wide range of environmental conditions. In humans, a pandemic H1N1 virus emerged in 2009 after the reassortment between a swine H1N1 virus from the Eurasian lineage and a swine H2N1 virus descended from a triple reassortment between a swine H1N1 virus from the North American lineage, an avian virus, and the human H3N2 virus. In aquatic birds, which are the main reservoir of these viruses, IAVs spread mainly by a fecal-oral route through water. In poultry such as chicken and turkeys, or in mammalian species, IAVs mainly have a respiratory tropism, and the virus spreads by contact between infected and susceptible hosts or by contaminated fomites, as well as through aerosols or respiratory droplets. In any case, the transmission routes of IAVs submit virus particles to a wide range of environmental conditions, which more or less rapidly affect them. In water, the time for IAV inactivation depends on widely studied abiotic factors such as temperature (Brown et al., 2007, 2009; Stallknecht and Brown, 2009; Nazir et al., 2010; Dublineau et al., 2011; Keeler et al., 2014; Zhang et al., 2014; Poulson et al., 2016), water salinity (Brown et al., 2009; Dublineau et al., 2011; Keeler et al., 2014; Poulson et al., 2016), and pH (Brown et al., 2009; Keeler et al., 2014; Poulson et al., 2016) and can range from a few days in saline water (35 g.L^-1^ NaCl) at 35°C to several years at 4°C (Dublineau et al., 2011). The environment acts both as a reservoir (Stallknecht et al., 2010; Roche et al., 2014) and as a bottleneck for IAVs’ evolution. It was experimentally demonstrated that IAV persistence in water is extremely variable among strains and subtypes (Stallknecht and Brown, 2009; Dublineau et al., 2011; Lebarbenchon et al., 2012). However, inactivation processes of avian IAVs in the environment are far from being well understood (Lebarbenchon et al., 2010). Only a few viral factors, such as the viral envelope origin and composition (Shigematsu et al., 2014; Bajimaya et al., 2017) and the pH of activation of the HA (Reed et al., 2010), are known to affect virus survival. On the other hand, the viral envelope and the viral genome are affected much more slowly than the actual loss of infectivity in water, suggesting that other viral factors drive IAV survival (Dublineau et al., 2011; Shigematsu et al., 2014). The envelope of IAV structure is made of two external glycoproteins, the hemagglutinin (HA) and the neuraminidase (NA), and a proton channel M2, which are inserted into a lipid bilayer surrounding a matrix M1 protein layer. Inside the viral particle, the segmented and negative strand RNA genome surrounded by a nucleocapsid (NP) interacts with the viral polymerase made of the three proteins PB1, PB2, and PA to constitute the ribonucleoprotein (RNP) complex. The eight RNPs interact closely with the M1 proteins.

In order to identify viral genetic drivers of the persistence phenotype, we generated reassortants from H1N1 viruses, which do not have the same persistence in an environmental model, and compared their inactivation slopes. As an environmental model, we used saline (35 g.L^-1^ NaCl) water at 35°C, because it allows observing differences of viral persistence more rapidly (Dublineau et al., 2011). Using fluorescence imaging microscopy, we also wanted to understand how our environmental model disrupts viral functions and infectivity of IAVs. We identified molecular determinants of the persistence phenotype in the environment, by introducing codon-optimized synonymous mutations or non-synonymous mutations in the HA or the NA gene. Altogether, our results demonstrate that the survival of Influenza viral particles is predominantly driven by the two external glycoproteins, and that environmental conditions affect the HA-mediated steps during viral entry.

## Results

### Influence of Viral Proteins on H1N1 Strains Persistence Outside the Host

**FIGURE 1:**
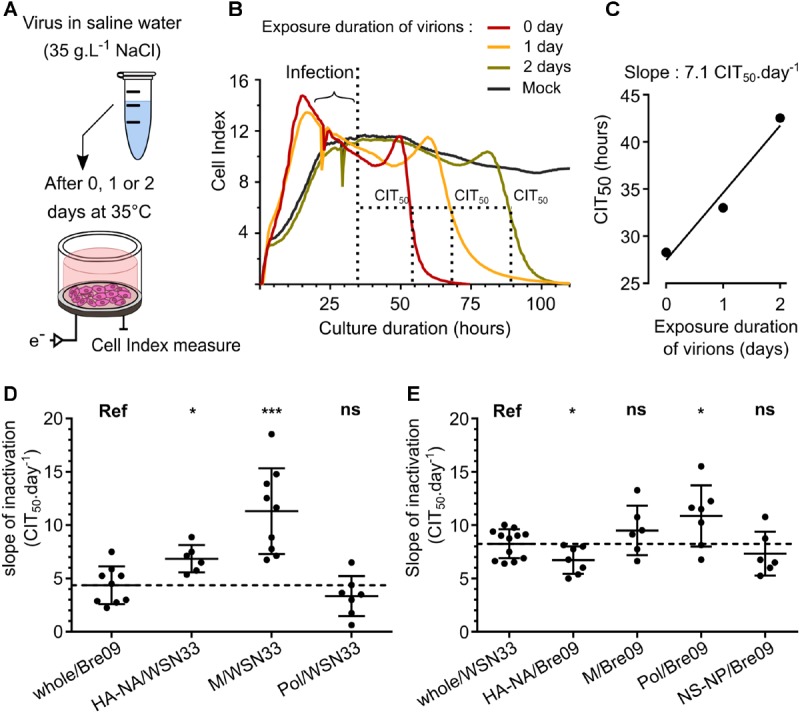
Influence of viral proteins on H1N1 viruses persistence in saline water at 35°C. **(A)** Viral particles were diluted in saline water (35 g.L^-1^ NaCl) and exposed to a temperature of 35°C for 0, 1, or 2 days. MDCK cells were infected by exposed viruses and impedance was monitored continuously and plotted as “cell index” (CI). **(B)** CI decrease due to virus-induced cytopathogenic effect was quantified with the CIT_50_ value, which is the necessary time in hours to measure a 50% decrease from the initial CI, always set as the CI value 5 h after infection (symbolized as a vertical dashed line). **(C)** The CIT_50_ values of an exposed virus increase with the duration of exposure in saline water at 35°C. It reflects the loss of infectivity and a linear regression analysis allowed calculating the viral inactivation slope. **(D,E)** Inactivation slopes comparison between **(D)** the 2009 wild-type H1N1 virus (reference; whole/Bre09) and reassortant viruses harboring different genomic segments of the 1933 H1N1 strain or **(E)** between the 1933 wild-type H1N1 virus (reference; whole/WSN33) and the reassortant viruses harboring genomic segments of the 2009 H1N1 strain. Means of inactivation slopes (horizontal lines) were compared using a Wilcoxon–Mann–Whitney test (ns, *P* > 0.1; ^∗^*P* < 0.05; ^∗∗∗^*P* < 0.0005). Each dot represents a single inactivation slope **(D,E)** and vertical lines represent the standard deviation.

Influenza A viruses, such as the seasonal H1N1 virus of 1999 and the pandemic H1N1 virus of 2009, show different persistence in water (Stallknecht and Brown, 2009; Dublineau et al., 2011; Lebarbenchon et al., 2012). This study aimed at characterizing viral molecular drivers associated with variations of persistence in saline water (35 g.L^-1^ NaCl) at 35°C used as an environmental model. These temperature and salinity conditions were selected because they allow observing variations between strains in a short period of time (Dublineau et al., 2011). In order to understand how viral proteins influence the persistence of H1N1 influenza particles, reassortant viruses between a pandemic H1N1 virus, the A/Bretagne/7608/2009 strain (whole/Bre09) and a pre-pandemic H1N1 virus, the A/WSN/1933 strain (whole/WSN33), were generated using a plasmid-based reverse genetics system. We rescued reassortant viruses containing either the HA/NA segments, the PB1/PB2/PA segments, the M segment, or the NS/NP segments from A/Bretagne/7608/2009 strain with the other segments from A/WSN/1933 as a complementary genomic backbone. We conversely rescued the same mirroring set of viruses with the same pool of segments but from the A/WSN/1933 strain and with the genomic backbone constituted by A/Bretagne/7608/2009 virus segments. The composition of each virus is detailed in **Tables 1, 2**. All reassortants grew except the NS-NP/WSN33 virus. Thus, we generated seven different reassortant viruses and compared their persistence with a real-time cell analysis system (RTCA), as described in the Methods section (**Figure 1A**). To quantify viral decay, we monitored CIT_50_ values after 1, 24, and 48 h of exposure in saline water at 35°C. The increase in CIT_50_ value over time reflects the loss of infectivity and the progressive inactivation of viral particles (**Figure 1B**). We calculated inactivation slopes from experimental CIT_50_ values (**Figure 1C**). The mean inactivation slope of the whole/Bre09 virus in saline water at 35°C was 4.4 CIT_50_.day^-1^ (**Figure 1D**), which is twice more stable than the whole/WSN33 virus that has a mean inactivation slope of 8.2 CIT_50_.day^-1^ (**Figure 1E**). When compared with the whole/Bre09 virus (**Figure 1D**), replacing the 2009 HA and NA by the 1933 HA and NA (HA-NA/WSN33 virus), or the 2009 M segment by a 1933 M segment (M/WSN33 virus) significantly destabilized the virus with mean inactivation slopes of 6.8 and 11.3 CIT_50_.day^-1^, respectively. On the contrary, the Pol/WSN33 virus persistence was not significantly different from that of the whole/Bre09 virus, with a mean inactivation slope of 3.3 CIT_50_.day^-1^. Compared with the whole/WSN33 virus (**Figure 1E**), replacement of the 1933 HA and NA by the 2009 HA and NA (HA-NA/Bre09 virus) significantly increased the persistence of the virus with a mean inactivation slope of 6.7 CIT_50_.day^-1^. The replacement of the 1933 M or NS and NP segments by their 2009 counterparts (M/Bre09 and NS-NP/Bre09 viruses) did not change significantly their persistence. More surprisingly, replacing the polymerase PB1, PB2, and PA segments of 1933 by the polymerase segments of 2009 (Pol/Bre09 virus) significantly destabilized this reassortant virus with a mean inactivation slope of 10.9 CIT_50_.day^-1^.

**Table 1:** Engineered reassortants between A/Bretagne/7608/2009 H1N1 virus and A/WSN/1933 H1N1 virus.

Reassortant name	A/Bretagne/7608/2009	A/WSN/1933
Whole/Bre09	HA, NA, M, PB1, PB2, PA, NS, NP	–
HA-NA/Bre09	HA, NA	M, PB1, PB2, PA, NS, NP
M/Bre09	M	HA, NA, PB1, PB2, PA, NS, NP
Pol/Bre09	PB1, PB2, PA	HA, NA, M, NS, NP
NS-NP/Bre09	NS, NP	HA, NA, M, PB1, PB2, PA
Whole/WSN33	–	HA, NA, M, PB1, PB2, PA, NS, NP
HA-NA/WSN33	M, PB1, PB2, PA, NS, NP	HA, NA
M/WSN33	HA, NA, PB1, PB2, PA, NS, NP	M
Pol/WSN33	HA, NA, M, NS, NP	PB1, PB2, PA


**Table 2:** Engineered reassortants between A/Paris/2590/2009 H1N1 virus and A/NewCaledonia/20/1999 H1N1 virus with the A/WSN/1933 H1N1 virus used as a genomic backbone.

Reassortant name	A/Paris/2590/2009	A/NewCaledonia/20/1999	A/WSN/1933
HA-NA/Par09	HA, NA	–	M, PB1, PB2, PA, NS, NP
HA-NA/Nc99	–	HA, NA	
HA_opti_-NA/Nc99	–	HA (codon optimized), NA	


Thus, once the HA and NA were isolated from their genomic context into a new virus, the persistence phenotype of this new virus tended to be the same as the virus from which HA and NA were originated. The matrix protein and to a certain extent the viral polymerase induced a decrease in viral persistence, showing that these segments, as well as different interactions between genes of specific constellations drive the environmental persistence of IAVs.

### Molecular Determinants of the HA Driving Environmental Persistence

**FIGURE 2:**
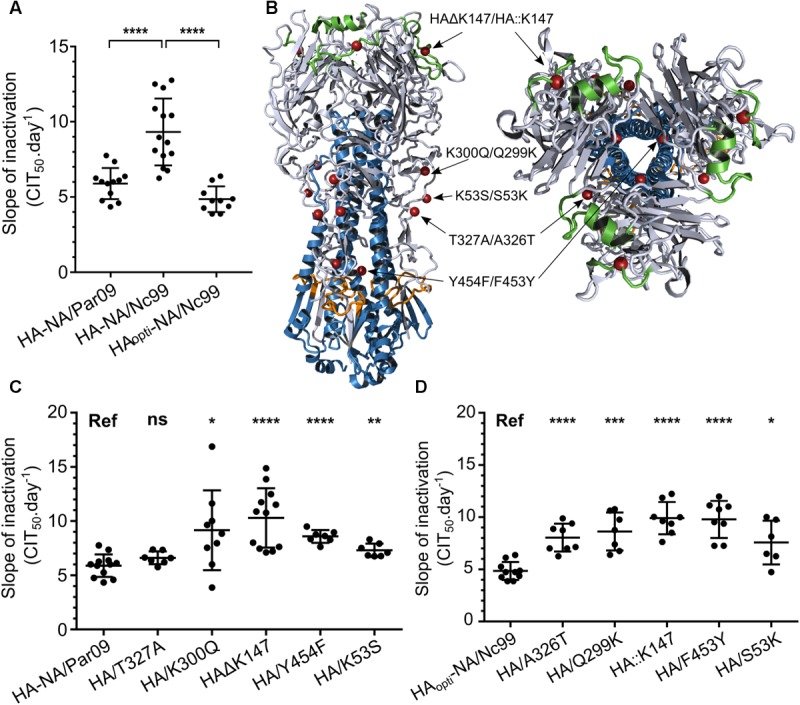
Synonymous and non-synonymous substitutions in the HA change environmental persistence. **(A)** Inactivation slopes of reassortant viruses bearing a HA and a NA either from 2009 (HA-NA/Par09), 1999 (HA-NA/Nc99), or 1999 with 24% of the HA mutated by synonymous substitutions (HA_opti-_NA/Nc99). Each dot represents a single inactivation slope and their means (horizontal lines) were compared using an ANOVA test (ns, *P* > 0.05; ^∗∗∗∗^*P* < 0.0001). **(B)** Selected mutations either on the HA of 2009 or the optimized HA of 1999 are indicated by red spheres and arrows on the crystal structure of a 2009 H1N1 virus trimeric HA (3LZG) (side or top-down views). **(C,D)** Mean inactivation slopes of mutated viruses were compared to the HA-NA/Par09 virus **(C)** or HA_opti-_NA/Nc99 virus **(D)**. Each dot represents a single inactivation slope value and their means (horizontal lines) were compared using a Wilcoxon–Mann–Whitney test (ns, *P* > 0.05; ^∗^*P* < 0.02; ^∗∗^*P* < 0.005; ^∗∗∗^*P* = 0.0001; ^∗∗∗∗^*P* < 0.0001). Vertical lines represent the standard deviation.

In order to understand how the HA protein modulates environmental persistence, we rescued two reassortant viruses bearing a HA and NA either from the H1N1 A/NewCaledonia/20/1999 strain (HA-NA/Nc99) or from the pandemic A/Paris/2590/2009 strain (HA-NA/Par09) (**Table 2**), both with the same genomic backbone belonging to the A/WSN/1933 strain. We selected these HA and NA because the two strains have a different persistence phenotype in saline water at 35°C (Dublineau et al., 2011) in favor of the pandemic strain. Using a third strain also allowed us to compare the HA and NA of the 2009 pandemic strain and the pre-pandemic strain independently of their parental genomic context. We wanted to assess whether introducing changes in the HA sequence had an impact on viral persistence. We thus synthesized a codon-optimized gene encoding the 1999 HA (HA_opti_), producing the same HA but containing 24.4% of synonymous substitutions. We then generated the corresponding reassortant virus bearing this HA_opti_ and the 1999 NA in the A/WSN/1933 backbone (HA_opti_-NA/Nc99; **Table 2**). The slope of inactivation was significantly lower in saline water at 35°C for the HA-NA/Par09 virus compared with the HA-NA/Nc99 virus inactivation slope (**Figure 2A**), respectively, 5.9 and 9.9 CIT_50_.day^-1^. This result is in agreement with that of a previous study (Dublineau et al., 2011) on the persistence phenotype of the wild-type non-reassortant A/Paris/2590/2009 and the A/NewCaledonia/20/1999 strains, and confirms that HA and NA are indeed the main viral factors leading the persistence of viral particles. Interestingly, HA_opti_-NA/Nc99 was as stable as HA-NA/Par09 and thus significantly more stable than HA-NA/Nc99 (**Figure 2A**) despite the identical amino acid composition of HA.

We also introduced non-synonymous mutations in the HA nucleotide sequence to study the impact of a single HA amino acid change on virus persistence. For this purpose, we analyzed the HA amino acid sequences of the HA-NA/Nc99 and HA-NA/Par09 viruses and, based on a preliminary mutation screening performed using a lentiviral pseudo-particles system (Sawoo et al., 2014), we selected five residues in the HA protein that might influence virus particle persistence outside the host (**Figure 2B**). Ten reassortant viruses bearing HA with amino acid substitutions or insertions/deletions were generated after site-directed mutagenesis of the pPol-HA plasmid. This insertion/deletion was selected because the 2009 HA possesses one more amino acid, at position 147, compared with the 1999 HA. We then exposed these mutated HA-NA/Par09 and HA_opti_-NA/Nc99 viruses to saline water at 35°C (**Figures 2C,D**). The HA/K300Q substitutions or HAΔK147 deletion in the HA of HA-NA/Par09 virus induced a significant increase in the mean inactivation slope, respectively, to 9.2 and 10.3 CIT_50_.day^-1^, thus generating very unstable mutants. Substitutions HA/Y454F and HA/K53S also destabilized the HA-NA/Par09 virus but to a lesser extent, whereas the HA/T327A substitution did not affect its persistence, with mean inactivation slopes of 8.6, 7.3, and 6.6 CIT_50_.day^-1^, respectively. The insertion of HA::K147 or substitutions A326T, F453Y, and Q299K in the HA of HA_opti_-NA/Nc99 virus greatly decreased the persistence of the virus, with a mean inactivation slope of 9.9, 8.0, 9.8, and 8.6 CIT_50_.day^-1^, respectively. The S53K substitution, however, had less impact on the persistence, with a mean inactivation slope of 7.6 CIT_50_.day^-1^. Altogether, these results demonstrated that synonymous mutations or a single amino acid change in the HA was sufficient to affect the viral persistence outside the host.

### Viral Particles Cannot Trigger Membrane Fusion After Being Exposed to Saline Water at 35°C

**FIGURE 3:**
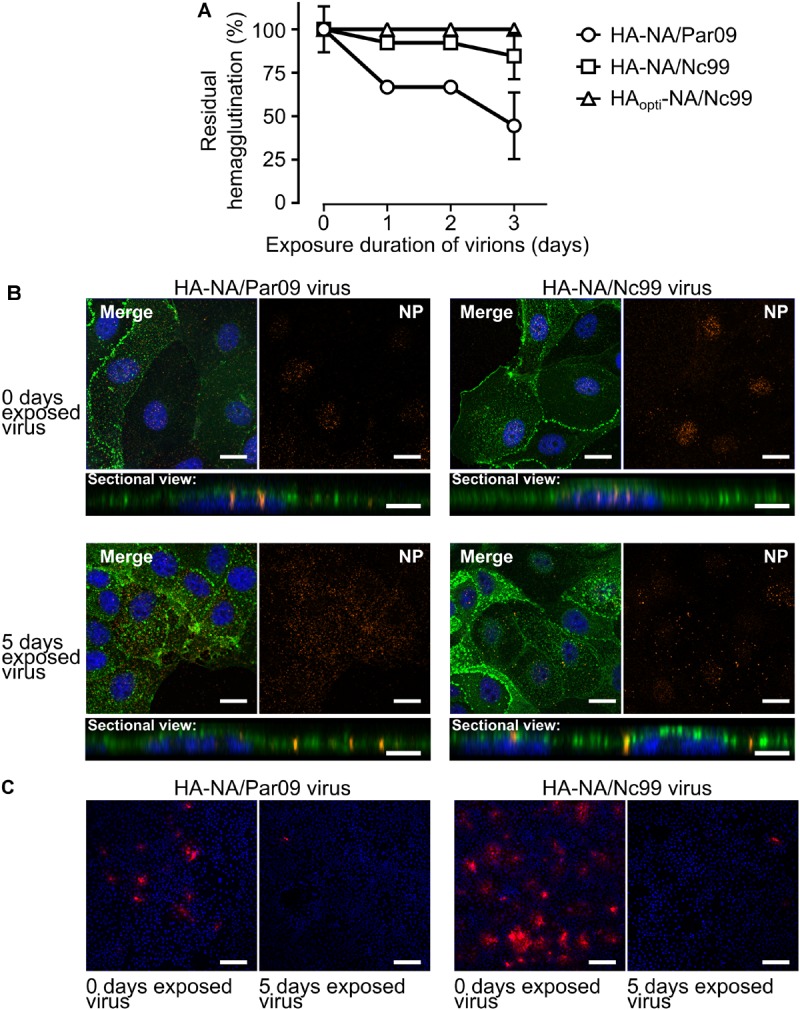
The HA of virus particles is still able to bind its cellular receptor but cannot trigger membrane fusion after being exposed. **(A)** Evolution of the residual hemagglutination titer (%) during exposure of reassortant viruses in saline water at 35°C. Vertical lines represent the standard deviation (*N* = 3). **(B)** Confocal immunofluorescence microscopy (×40) of **(A)** MDCK cells 2 h after infection by either the HA-NA/Par09 virus (left) or the HA-NA/Nc99 virus (right), which have been exposed (bottom) or not (top) for 5 days to saline water (35 g.L^-1^ NaCl) at 35°C. Signal of the influenza NP immunolabeling is shown in orange. WGA labeling signal at the cell membrane is shown in green and the nucleus labeling with Hoescht dye is shown in blue. For each condition a merge of all channels (top left), the NP immunolabeling acquisition channel only (top right) or a Z-stack of sectional views at a distance of 0.42 μM (bottom) are shown. **(C)** MDCK cells (×10 magnification) 20 min after infection with both HA-NA/Par09 and HA-NA/Nc99 viruses previously labeled with the R18 fluorophore at a self-quenched concentration. R18 dequenching signal due to the fusion of the viral envelope with the endosomal membrane is shown in red. Scale bars represent 20 μm (**B**, merged and NP views), 10 μm (**B**, sectional views), and 100 μm **(C)**.

Since the loss of hemagglutination titer of viral particles is slower than their loss of infectivity (Chu, 1948) (**Figure 3A** and Supplementary Figure S1), we decided to evaluate whether viruses were still able to bind their cellular receptor. For this purpose, we immuno-labeled the viral nucleoprotein (NP), which encapsidates the viral genome to form the ribonucleoprotein, and used confocal microscopy for its detection in infected MDCK cells. In cells infected with non-exposed HA-NA/Par09 or HA-NA/Nc99 viruses, the NP protein was concentrated within the nucleus 2 h after infection (**Figure 3B**). On the contrary, in MDCK cells infected with exposed HA-NA/Par09 or HA-NA/Nc99 viruses for 5 days to saline water at 35°C, we detected the NP protein close to the cell membrane but not in the nucleus after 2 h of infection. We confirmed that NP localization of exposed viruses was similar to that of non-exposed viruses after 20 min of infection, when virus entry is not completely achieved (Supplementary Figure S2). We thus assessed the HA-triggered fusion of the viral membrane in infected MDCK cells using R18-labeled viruses at self-quenched concentration, a widely used technique to detect fusion of the viral membrane with the endosomal membrane (Pohl et al., 2014; Schelker et al., 2016). Fusion events were detected 20 min after infection by confocal microscopy in cells infected with viruses exposed to saline water for 5 days at 35°C or non-exposed viruses (**Figure 3C**). We detected almost no R18 dequenching signal in cells infected with HA-NA/Par09 or HA-NA/Nc99 exposed viruses, whereas we observed numerous fusion events in cells infected with the non-exposed viruses. This result demonstrates that the loss of infectivity of H1N1 viruses in saline water at 35°C is a consequence of the HA inability to trigger membrane fusion.

### HA Stability at Low pH Is Not the Only Determinant of Viral Persistence in Water

**FIGURE 4:**
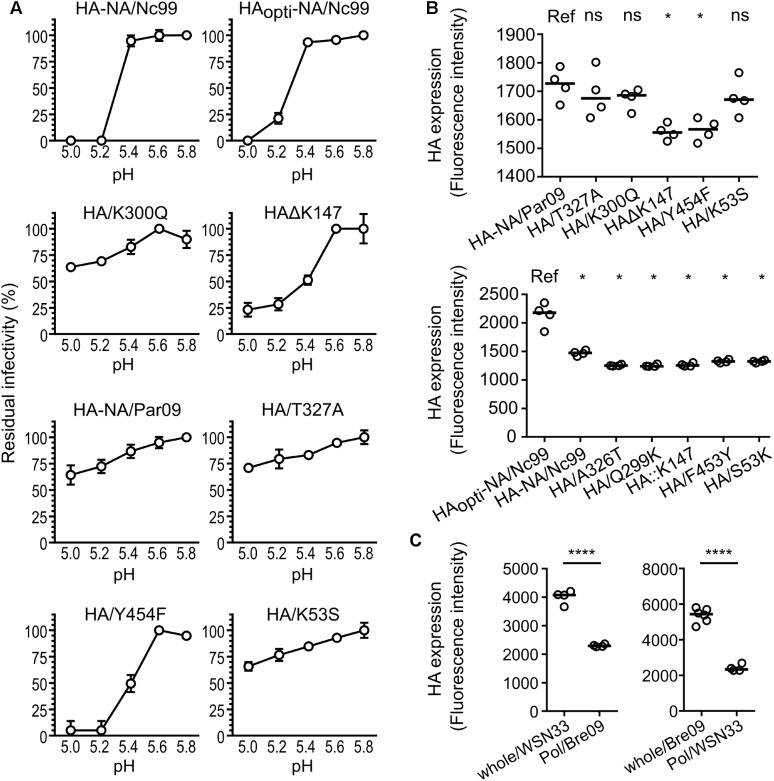
Virus stability to lower pH exposure and HA expression in infected cells. **(A)** H1N1 reassortant viruses either with a 1999 HA (wild-type or optimized) or a 2009 HA (wild-type or with a single amino acid substitution) were exposed to pH-adjusted PBS buffer for one hour and the residual infectivity values (in percentage from the initial TCID_50_ titer) were calculated. Spheres represent the mean residual infectivity and vertical lines represent the standard deviation (*N* = 3). **(B,C)** HA surface expression in infected MDCK cells analyzed by flow cytometry detecting Alexa Fluor 488 signal 24 h after infection. Horizontal lines represent the median of fluorescence intensities and HA surface expression levels were compared using a Wilcoxon–Mann–Whitney test (ns, *P* > 0.05; ^∗^*P* < 0.02; ^∗∗∗∗^*P* < 0.0001).

A link between HA stability at low pH and stability to increasing temperatures has already been proposed (Krenn et al., 2011; Imai et al., 2012). Therefore, we measured the persistence of HA-NA/Par09, HA-NA/Nc99, and mutated viruses in various pH-adjusted PBS solutions for 1 h at room temperature (**Figure 4A**). In addition, we quantified HA cell surface expression by flow cytometry in MDCK cells at 24 h post-infection (**Figure 4B**). The most unstable viruses (HA/Y454F and HAΔK147 viruses; **Figure 2C**) showed a lower HA surface expression level (**Figure 4B**) and had a higher sensitivity at low pH compared with HA-NA/Par09 virus. For the stable HA_opti_-NA/Nc99 virus, we observed a higher HA surface expression level compared with the unstable HA-NA/Nc99 virus, but they both had a high sensitivity at low pH. Similarly, the whole/WSN33 virus, which was more stable than the Pol/Bre09 virus (**Figure 1E**), even if they both have the same HA and NA, had much higher HA expression levels in infected cells (**Figure 4C**). Altogether, our results support the idea that HA expression level is a viral driver of environmental persistence when the HA has a high pH of inactivation.

### Molecular Determinants of the NA Driving Environmental Persistence

**FIGURE 5:**
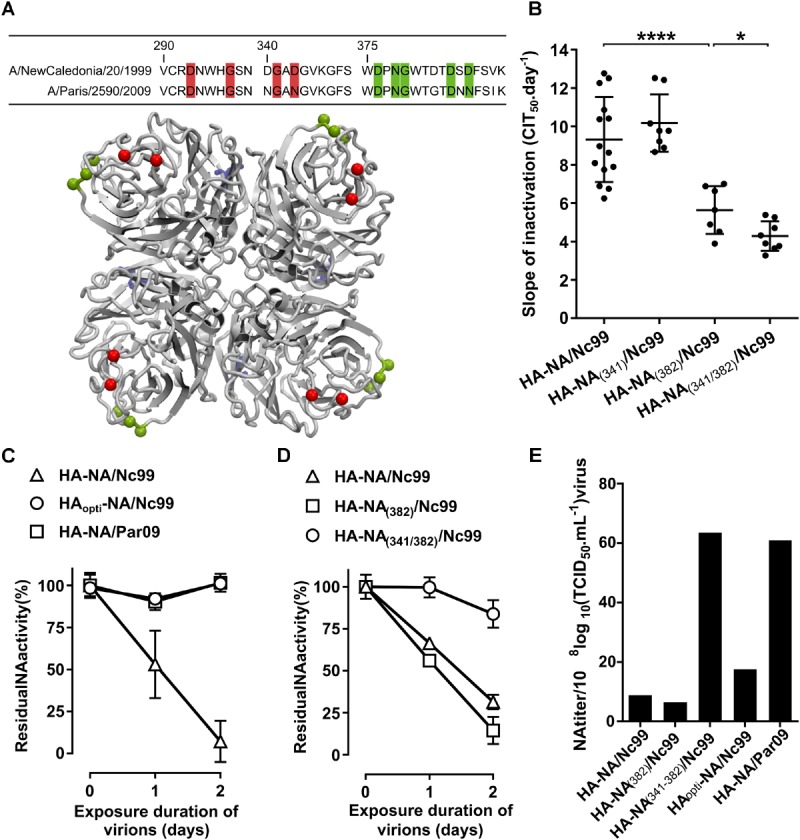
Non-synonymous substitutions in the NA change environmental persistence. **(A)** Alignment of amino acid sequences of NA from 1999 and 2009 H1N1 viruses. Putative amino acids involved in the second (red) and third (green) calcium-binding site are highlighted. Selected mutations on the 1999 NA are indicated by red (NGAN/341/DGAD) or green (GTDNN/382/DTDSD) spheres on the crystal structure of a 2009 H1N1 virus tetrameric NA (4B7Q) (top-down view). **(B)** Mean inactivation slopes of viruses after exposure to saline water at 35°C. Each dot represents a single inactivation slope and their means (horizontal lines) were compared using an ANOVA test (^∗^*P* < 0.05; ^∗∗∗∗^*P* < 0.0001). **(C,D)** Evolution of residual NA activity (%) during exposure of reassortant viruses in saline water at 35°C. Panels **(C,D)** represent two individual experiments. Vertical lines represent the standard deviation (*N* = 3). **(E)** NA titer of reassortant viruses measured with a twofold serial dilution steps and then calculated for viral titer of 8 log_10_TCID_50_.mL^-1^ (*N* = 2).

Based on the results obtained with our reassortant viruses, the NA might be as important as the HA in driving the persistence phenotype (**Figures 1D,E**). Previous studies have shown that the NA stability is strain dependent, and is linked to the calcium binding by the protein (Baker and Gandhi, 1976; Burmeister et al., 1994). The 2009 pandemic NA has three calcium-binding sites (Air, 2012), as well as the 1918 NA (Xu et al., 2008), and thus presumably the 1999 NA. After alignment of the 1999 and 2009 NA protein sequences (**Figure 5A**), we observed differences in the amino acids directly involved in the second and the third calcium-binding sites (Air, 2012), respectively, near the active site and the side chains (D381, D387, and D379) (**Figure 5A**). We then decided to substitute the amino acids around the positions DGAD/341/NGAN (substitutions D341N and D344N), and/or DTDSD/382/GTDNN (substitutions D382G, S385N, and D386N) (**Figure 5A**). We generated reassortant viruses with a mutated NA either in position 341 (HA-NA_(341)_/Nc99 virus), or in position 382 (HA-NA_(382)_/Nc99 virus) or in both positions (HA-NA_(341/382)_/Nc99 virus), all with a 1999 HA and a genomic backbone from the 1933 H1N1 virus. We determined the persistence of these mutant viruses in saline water at 35°C by measuring a mean inactivation slope of 10.2 CIT_50_day^-1^ for the HA-NA_(341)_/Nc99 virus, 5.6 CIT_50_.day^-1^ for the HA-NA_(382)_/Nc99 virus, and 4.3 CIT_50_.day^-1^ for the HA-NA_(341/382)_/Nc99 virus. In comparison, the HA-NA/Nc99 virus had a mean inactivation slope of 9.9 CIT_50_.day^-1^ (**Figure 5B**). We thus observed a positive effect of mutations at the third calcium-binding site toward long-lasting persistence for the HA-NA_(382)_/Nc99 virus. A cumulative effect of mutations at both calcium-binding sites was also observed, leading to a significant increase in HA-NA_(341/382)_/Nc99 virus survival compared with HA-NA_(382)_/Nc99 virus. The results demonstrated that amino acids at the calcium-binding sites in the NA are important molecular determinants of virus environmental persistence. The HA-NA_(341-382)_/Nc99 virus, which had the highest environmental persistence (**Figure 5B**) also had a stable NA activity (**Figure 5D**). This link was, however, not observed for the HA-NA_(382)_/Nc99 virus, with an intermediate environmental persistence. Similarly, we observed that HA_opti_-NA/Nc99 and HA-NA/Par09 viruses, which had a mean inactivation slope below 6 CIT_50_.day^-1^, also presented a higher NA activity compared with HA-NA/Nc99 virus. Unfortunately, the NA activity for the HA-NA_(341)_/Nc99 virus was below our detection threshold and could not be measured.

## Discussion

Differences in environmental persistence among influenza virus strains of the same subtype were previously described (Stallknecht and Brown, 2009; Dublineau et al., 2011; Lebarbenchon et al., 2012), without providing a clear molecular basis to this phenomenon. It was also demonstrated that their viral genome was not degraded and that the viral envelope remained intact after a few days in saline water at 35°C (Dublineau et al., 2011; Shigematsu et al., 2014). The results produced in our controlled experimental model with H1N1 viral strains are the first to describe molecular basis of the IAVs persistence outside the host. Comparing the persistence of multiple engineered reassortants allowed us to identify the HA and NA proteins as major viral drivers of the virus survival outside the host (**Figures 1D,E, 2A**).

Codon optimization of the HA gene increased considerably the viral persistence of the HA_opti_-NA/Nc99 virus compared with the non-optimized HA-NA/Nc99 virus (**Figure 2A**) while they both harbored an identical HA protein. Codon usage bias that favors synonymous mutations of HA exists in nature, particularly at the more functionally constrained residues (Plotkin and Dushoff, 2003). It would be interesting to investigate whether the viral codon-optimization in nature also confers adaption of the persistence phenotype to the environment. In addition, we found amino acid changes in the HA that affected the persistence of the whole virus in our environmental model. The two mutations responsible for the greatest decrease in viral persistence were located near the receptor binding site (position 147) and in the internal stem (position 454 or 453), while the others were located in the external loops of the HA1 subdomain (**Figure 2B**). Some mutations affecting HA stability in low pH conditions were previously described (Byrd-Leotis et al., 2015; Russier et al., 2016) and few mutations were associated with a higher persistence outside the host (Reed et al., 2010) and to heat inactivation (Krenn et al., 2011). Similarly, we observed a link between pH stability of our 2009 HA-mutated viruses (**Figure 5A**) and their persistence in water.

Our results also demonstrated that a virus with a high sensitivity to low pH still has the potential to be stable in the environment. Indeed, HA-NA/Nc99 and HA_opti_-NA/Nc99 viruses both displayed high sensitivity to low pH although they have a very different persistence phenotype in water. We also observed a higher HA surface expression level in cells infected with HA_opti_-NA/Nc99 virus (**Figure 4B**), in agreement with a previous study using codon optimized HA (Jiang et al., 2007). Altogether, our results suggest that the survival likelihood for a virus increases when its HA surface level is higher, warranting to examine further the correlation between HA level at the cell surface and HA level at the virion surface, as demonstrated for pseudo-particles bearing H3 proteins (Hsu et al., 2016). Results obtained with the Pol/Bre09 and the whole/WSN33 reassortant viruses, for which we expected different transcription and replication efficiencies of the HA segment, are in agreement with this finding (**Figures 1E, 4C**). Although HA-NA/Bre09 and HA-NA/WSN33 are, respectively, more stable and less stable, compared with their parental whole/WSN33 and whole/Bre09 viruses, they have the same inactivation slope (**Figures 1D,E**). Different replication efficiencies of the polymerase complexes resulting in various HA expression levels might explain this observation.

Similarly, the less stable HAΔK147 and HA/Y454F mutant viruses (**Figure 4A**) have a high sensitivity to low pH and lower HA expression levels in infected cells compared to the other mutants (**Figures 4A,B**). On the other hand, the whole/Bre09 and the Pol/WSN33 viruses had the same persistence but significant differences of HA expression levels (**Figure 4C**). They both harbor the same HA of 2009, which is stable at low pH. Overall, these results suggest that the persistence of a virus with a HA resistant to low pH does not vary with HA expression level.

The mutation HA/K300Q, which is not located in the fusion peptide and is far from the pH sensor peptide, does not significantly affect the sensitivity of the HA at low pH, but affects virus stability in saline water. The glutamine is a more reactive and less stable amino acid than the lysine, since it has a shorter carbon chain and a hydroxyl functional group at the end of the chain. A calculation of the surface potential electrostatic also revealed that the lysine in position 300 is the only negatively charged amino acid in the middle of a local neutral patch. The replacement of this lysine changes the surface electrostatics of this patch to a slight negatively charged patch that could explain the stability phenotype observed for this mutant (Strickler et al., 2006).

After losing their infectivity, HA-NA/Par09 and HA-NA/Nc99 viruses were still able to attach to cells but could not trigger viral fusion after endocytic uptake. Three or four neighboring HA trimers are probably mandatory to reach the hemifusion step occurring during the fusion process (Ivanovic et al., 2013). This number might not be reached for viruses that do not have a sufficient density of functional HA at their surface and could explain the observed absence of fusion after exposure to a hostile environment. This observation might in turn provide an explanation for how viruses such as HA_opti_-NA/Nc99, with higher HA surface levels stay stable longer, as the HA inactivation is probably progressive in the environment as suggested by our results on the loss hemagglutination activity (**Figure 3A**). In this assay, the loss of hemagglutination capacity of exposed viruses does not perfectly reflects their slopes of inactivation (**Figures 2A, 3A**). This may be explained by the fact that HA-NA/Nc99 and HA_opti_-NA/Nc99 viruses, respectively, possess a very unstable NA (**Figure 5C**) and a higher HA expression surface (**Figure 4B**). These two factors could contribute to the higher stability of their hemagglutination capacity during exposure to the environment.

We provided evidences that the NA protein is also a driver of IAVs environmental persistence. Our results highlighted the importance of amino acid positions in the calcium-binding sites for both NA stability and virus survival. It was previously shown that the NA from the 2009 and 1918 H1N1 pandemic viruses possess a third calcium-binding site coordinated by side chains (Xu et al., 2008; Air, 2012), suggesting that all the human N1 may contain three calcium-binding sites. Based on a sequence alignment between the NA of the 2009 and 1999 H1N1 viruses and observations of the structural features, we substituted key amino acids involved in the second and the third calcium-binding sites. These mutations induced an important increase in the environmental persistence of both the HA-NA_(382)_/Nc99 and HA-NA_(341/382)_/Nc99 viruses compared with the HA-NA/Nc99 virus (**Figure 5B**). Our results raised new questions on this putative third calcium-binding site in the human N1, which may not be present or possess a weaker affinity for calcium ions in the NA of the pre-pandemic H1N1 of 1999. Moreover, we observed that the more stable virus in the environment, such as HA_opti_-NA/Nc99 virus and the double mutant HA-NA_(341/382)_/Nc99 virus, induced a high HA surface expression level or had a stable and high NA activity (**Figures 4B, 5**). The mutations introduced in the HA or the NA probably modified the viral HA/NA balance, which might be an important molecular determinant of environmental persistence, possibly by increasing sialic acid binding, which was suggested to stabilize the influenza virus (Souris et al., 2015; Hirose et al., 2017). These results raised new questions about the role of the NA protein in the viral entry, which is not yet fully understood.

The introduction of the M segment from A/WSN/1933 virus into the A/Bretagne/7608/2009 virus (M/WSN33 virus) induced a significant decrease in the mean inactivation slopes (**Figure 1D**). This result raised questions on the role of the matrix protein on the environmental persistence of IAVs. The matrix protein is a factor involved in determining viral particle morphology, with effects on the HA stability as well as the neuraminidase activity (Campbell et al., 2014; O’Donnell et al., 2014).

Based on previous published results, we found that a log-linear model drives influenza A virus inactivation in our experimental model (Dublineau et al., 2011). We observed that the distributions of the inactivation slopes among the different viruses were significantly different (Bartlett’s statistic, *P* < 0.05), so we used a non-parametric Wilcoxon–Mann–Whitney test to compare virus persistence. Moreover, we observed that the standard deviation of the mean inactivation slopes tends to increase in the experiments performed with low persistence viruses compared with experiments with high persistence viruses (Supplementary Figure S3). It is possibly because of minor genetic variants or morphological variants, present in every viral population, which could have a higher persistence than the average population. Minor variants proportion may increase during the viral decay monitoring of low persistence viruses. Indeed, the average population of high persistence viruses remained infectious for a longer period and is more homogeneous after a long time at 35°C compared with low persistence viruses that are submitted to a more important bottleneck.

The putative role of particle persistence in the environment for IAV transmission has been discussed in reviews (Weber and Stilianakis, 2008; Sooryanarain and Elankumaran, 2015). Using computational approaches, it was suggested that the virus persistence in the environmental reservoir is an important parameter impacting overwintering of IAVs, infection probability of migratory ducks in low density population areas as well as spatial variations of IAV spreading (Lang et al., 2008; Roche et al., 2014; Vittecoq et al., 2017). IAVs persistence could also explain their evolutionary dynamic. It was shown that hemagglutinin diversity found in avian IAVs is positively correlated to viral persistence (Roche et al., 2014). This could allow viral strains to adapt to local environmental conditions (Lebarbenchon et al., 2012). In addition, evidence of IAV persistence was shown in Antarctica (Hurt et al., 2016) and in Siberian ice lakes (Zhang et al., 2006) where viruses are most likely stable for years (Brown et al., 2007; Dublineau et al., 2011), justifying to further investigate the role of long-term persistence on virus reintroduction. The present study provides for the first time experimental data highlighting the role of the HA variability in driving influenza persistence in the environment.

We chose saline water (35 g.L^-1^) at 35°C as an environmental model. This salinity is the average salt concentration in the ocean. Even if a large part of the seawater surfaces on earth has a temperature between 20 and 35°C, this model cannot be representative of all environmental conditions met by IAV particles. Studying identified molecular drivers of environmental viral persistence at lower temperatures would be interesting to address in the future.

## Conclusion

In conclusion, the molecular drivers of influenza virus persistence that we identified in the present study could help in refining ecological model of IAVs transmission and their genetic diversity in the environment. Our results are providing an experimental basis to further investigate the role of the HA and NA proteins in driving the phenotype of persistence among all influenza viral subtypes and establish the impact of their persistence on viral transmission in the environment.

## Materials and Methods

### Cells and Viruses

Madin–Darby canine kidney epithelial (MDCK) cells were maintained in Modified Eagle’s Medium (MEM) (GIBCO, Thermo Fisher Scientific), supplemented with 10% fetal calf serum (FCS) and antibiotics (100 units.mL^-1^ penicillin, 100 mg.mL^-1^ streptomycin, GIBCO, Life Technologies). Human embryonic kidney cells 293T (HEK-293T) were maintained in Dulbecco’s Modified Eagle’s medium (DMEM) (GIBCO, Thermo Fisher Scientific) supplemented with 10% FCS. All cells were incubated at 37°C in humidified 5% CO_2_ incubator. MDCK cells were infected at a multiplicity of infection (MOI) of 10^-4^ plaque-forming units per cell (pfu.cell^-1^) and maintained in MEM without FCS in the presence of 1 μg.mL^-1^ of TPCK-trypsin (trypsin/L-1-tosylamide-2-phenylethyl chloromethyl ketone; Worthington Biochemical Corporation) at 35°C for 3 days to generate stock viruses. The clarified supernatants were harvested, aliquoted, and stored at –80°C.

### Generation of Reassortant Viruses and Mutated Viruses

Recombinant viruses were rescued after co-transfection of MDCK and 293T co-cultivated cells using FuGENE HD transfection reagent (Promega) at a ratio of 3:1 (μL:μg). A/WSN/1933 recombinant virus was rescued by reverse genetics using a twelve plasmids transfection system, with eight pPol transcription plasmids containing an individual genomic segment under the control of a truncated human RNA polymerase I promoter and upstream the hepatitis Delta virus ribozyme as well as four pCDNA expression plasmids containing either the NP, PA, PB1, or PB2 gene under the control of a cytomegalovirus promoter as described previously (Fodor et al., 1999). A/Bretagne/7608/2009 recombinant virus was rescued by reverse genetics using an eight plasmids transfection system as described previously (Hoffmann et al., 2000). Reassortant viruses between A/WSN/1933 and A/Bretagne/7608/2009 strains were rescued using plasmids from those two reverse genetics systems (reassortant viruses are listed in **Table 1**). Reassortant viruses harboring HA and NA from A/Paris/2590/2009 or A/NewCaledonia/20/1999 strains with the internal genes of A/WSN/1933 were rescued using the twelve plasmids transfection system for A/WSN/1933 (reassortant viruses are listed in **Table 2**). Nucleotide changes were introduced into the pPol-HA plasmid by using the QuikChange II site-directed mutagenesis kit (Agilent) in accordance with the manufacturer’s instructions. Because the pPol-HA plasmid expressing the A/NewCaledonia/20/1999 HA segment was unstable in bacteria, the HA DNA sequence was replaced by a codon-optimized sequence and used for transfection. Virus identity and absence of unintended mutations were confirmed by Sanger sequencing using BigDye Terminator v1.1 cycle sequencing kit (Applied Biosystems) and a 3730 DNA Analyzer (Applied Biosystems).

### Virus Persistence in Saline Water at 35°C

Virus persistence in saline water at 35°C was studied as follows: viral suspensions were diluted 10 times in saline distilled water (35 g.L^-1^ NaCl) and placed in a humidified incubator (5% CO_2_, 35°C) for 1, 24, or 48 h. The pH of the saline water did not vary between experiments. In order to quantify their residual infectivity, MDCK cells were seeded on a 16-well microtiter plate (30,000 cells per well) coated with microelectrode sensors in the xCELLigence^®^ Real-Time Cell Analysis (RTCA) DP instrument (ACEA Bioscience, Inc.) and grown for 24 h (5% CO_2_, 37°C). Cells were then infected by exposed viruses and cell impedance, expressed as an arbitrary unit called the cell index (CI), was measured through the electrodes every 15 min. CI decrease due to virus-induced cytopathogenic effect was quantified with the CIT_50_ value, which is the necessary time in hours to measure a 50% decrease from the initial CI, always set as the CI value 5 h after infection. CIT_50_ values are linearly correlated to the TCID_50_ titers of the viral suspensions (Fang et al., 2011) (Supplementary Figure S4). Thus, the increase of the CIT_50_ values over time reflects the loss of infectivity. For each exposed viral suspension, CIT_50_ values were obtained at different exposure times and plotted to calculate a linear regression slope referred to as the inactivation slope. All data shown on viral persistence have been obtained at different periods and with different viral stock productions. Each replicate value of the inactivation slopes refers to a replicate virus suspension tested for persistence.

### Virus Concentration for Functional Analyses

For the experiments described below, harvested supernatants of stock viruses were clarified and concentrated using a vivaspin 20 centrifugal concentrator (1,000,000 MWCO, Sartorius), to reduce the initial volume by 10 times. Concentrated supernatants were then diluted in saline distilled water (35 g.L^-1^ NaCl) at a ratio of 1:10 and placed either at 4°C (0 day exposure) or at 35°C for 5 days and then kept at 4°C until used in further analysis.

### Hemagglutination Assay

Exposed viruses were diluted in twofold dilution steps with PBS in a 96-wells plate and mixed with an equal volume of a 0.75% suspension of fresh guinea pig erythrocytes. The mixture was incubated for 1 h at room temperature before observing erythrocytes aggregation.

### Neuraminidase Activity Assay

Neuraminidase activity of exposed viruses was measured using a NA-Fluor kit (Applied Biosystems) according to manufacturer’s protocol. Viruses were diluted in twofold dilution steps and mixed with an equal volume of MUNANA (2′-(4-methylumbelliferyl)alpha-D-*N*-acetylneuraminic acid) substrate. After 1 or 2 h, the level of fluorescence was quantified with a microplate fluorimeter (TriStar^2^ LB942, Berthold).

### Fluorescence Microscopy

For influenza virus nucleoprotein (NP) labeling, MDCK cells were seeded on glass coverslips in 6-well plates for 24 h before inoculation with exposed viruses diluted 1:10 in MEM. Twenty minutes or 2 h post-infection, cells were washed twice with MEM and fixed with paraformaldehyde 2% (w/v) for 10 min. Cell membranes were labeled using Alexa Fluor 488 conjugated wheat germ agglutinin (Thermo Fisher Scientific) at 4 μg.mL^-1^ for 10 min and permeabilized during 20 min in PBS buffer containing 0.2% Triton X-100. Fixed cells were incubated at 4°C overnight in the presence of an anti-NP antibody (MAB8257, Merck Millipore) diluted in PBS buffer containing 2% BSA at a concentration of 1 μg.mL^-1^, then incubated at room temperature for 2 h with an Alexa Fluor 647 F (ab′)2-Goat anti-Mouse IgG (H+L) Cross-Adsorbed Secondary antibody (Thermo Fisher Scientific) diluted at a concentration of 2 μg.mL^-1^. For Influenza virus envelope labeling and endosomal fusion analyses, concentrated viral suspensions were incubated in the presence of rhodamine B (R18, Thermo Fisher Scientific) and Dioc_18_ (3) (3,3′-dioctadecyloxacarbocyanine perchlorate, Thermo Fisher Scientific) for 2 h at room temperature both at a final concentration of 20 μM. Viral suspensions were then applied on a PD-10 desalting column (GE Healthcare) using gravity flow according to the manufacturer’s instructions to remove fluorophore in suspension. MDCK cells seeded on glass coverslips in 6-well plates for 24 h were placed at 4°C for 5 min prior to infection in order to synchronize viral fusion (Schelker et al., 2016) and were then inoculated with labeled viral suspensions. After infection, the cells were incubated at 35°C for 20 min and then washed twice with MEM and fixed with paraformaldehyde 2% (w/v) for 10 min. In all experiments, cell nuclei were labeled using Hoescht 33342 diluted in PBS at a concentration of 5 μg.mL^-1^ and incubated for 10 min. Glass coverslips were then mounted on a glass slide with Prolong gold antifade reagent (Thermo Fisher Scientific). Confocal laser scanning of fluorescence was performed using LSM700 inverted microscope (Zeiss) equipped with a plan apochromat 10× objective with a numerical aperture (N.A) of 0.45 and an enhanced contrast plan-neofluar 40× oil objective with a N.A of 1.3.

### HA Stability to pH

Three independent virus replicates were diluted in a pH-controlled PBS solution (at a resolution of 0.2) with citric acid and incubated 1 h at room temperature. Residual infectivity of each replicate was then quantified by a TCID_50_ method, as described previously (Dublineau et al., 2011).

### HA Expression Level by Flow-Cytometry

Cell surface HA expression of infected cells was measured by immunofluorescence using flow cytometry. MDCK cells were infected for 24 h in 6-well plates at a MOI of 0.5 pfu.cell^-1^ in the presence of TPCK-trypsin. Cell monolayers were washed twice with PBS, re-suspended using trypsin-EDTA (Thermo Fisher Scientific), fixed with paraformaldehyde 2% (w/v) for 10 min, and incubated with an anti-HA monoclonal antibody (Sinobiological) at a concentration of 1 μg.mL^-1^ at 4°C overnight. Cells were then incubated for 30 min at room temperature in the presence of a species-specific Alexa Fluor 488 conjugated secondary antibody (Thermo Fisher Scientific). After centrifugation, cells were re-suspended in PBS and analyzed with an Attune NxT Flow Cytometer (Thermo Fisher Scientific). For each replicate, 50,000 cells were analyzed with the same parameters.

### Statistical Analyses and Software

Numerical data were analyzed with Prism software version 6.07 (Graph Pad Software). If needed, a Grubb’s test was performed to identify and remove outliers. ANOVA tests were performed for statistical comparison when variances were equal across samples (Bartlett’s test). Otherwise a two-tailed, Wilcoxon–Mann–Whitney test, was used to compare samples distribution. DNA sequence alignments and Sanger sequencing analysis were performed using CLC Main Workbench version 7.7.3 (Qiagen). Hemagglutinin structure was visualized using the PyMOL Molecular Graphics system version 1.8 (Schrodinger, LLC). Microscopy imaging data were processed using LSM software Zen Blue edition version 2.3 (Zeiss) and flow cytometry data were analyzed with FlowJo (LLC) software.

## Data Availability

The raw data supporting the conclusions of this manuscript will be made available by the authors, without undue reservation, to any qualified researcher.

## Author Contributions

TL conceived, carried out, and analyzed all the experiments. CB provided materials and assistance. TL, IL, and J-CM wrote the manuscript. IL and J-CM supervised the project.

## Conflict of Interest Statement

The authors declare that the research was conducted in the absence of any commercial or financial relationships that could be construed as a potential conflict of interest.
