# Supplementary material for: Influenza Virus Segment Composition Influences Viral Stability in the Environment
Source: Front Microbiol. 2018 Jul 9;9:1496. doi: 10.3389/fmicb.2018.01496 (PMC6046443; doi:10.3389/fmicb.2018.01496)
Supplement: Supplementary file 1 [file Data_Sheet_1.pdf]

Supplementary Material

Influenza virus segment composition influences viral stability in the environment

Thomas Labadie [*], Christophe Batéjat, Jean-Claude Manuguerra, India Leclercq [*]

*** Correspondence:** thomas.labadie@pasteur.fr, india.leclercq@pasteur.fr

1. Supplementary figures

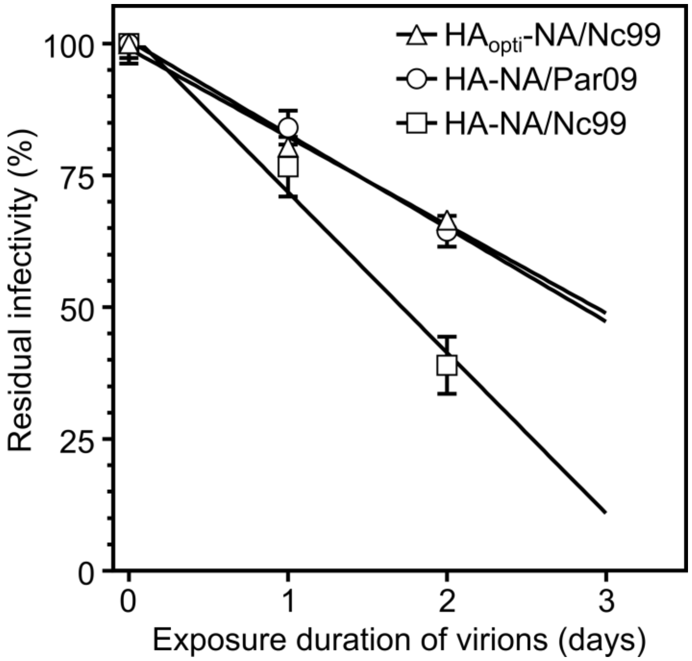

Supplementary Figure 1. Residual infectivity of exposed viruses to saline water at 35°C over time. Evolution of the residual CIT_{50} over time for HA-NA/Par09, HA-NA/Nc99 and HA_{opti}-NA/Nc99 viruses. For each virus, a linear regression slope of the residual infectivity data is shown (solid line) as well as the standard error for each time point (N=10).

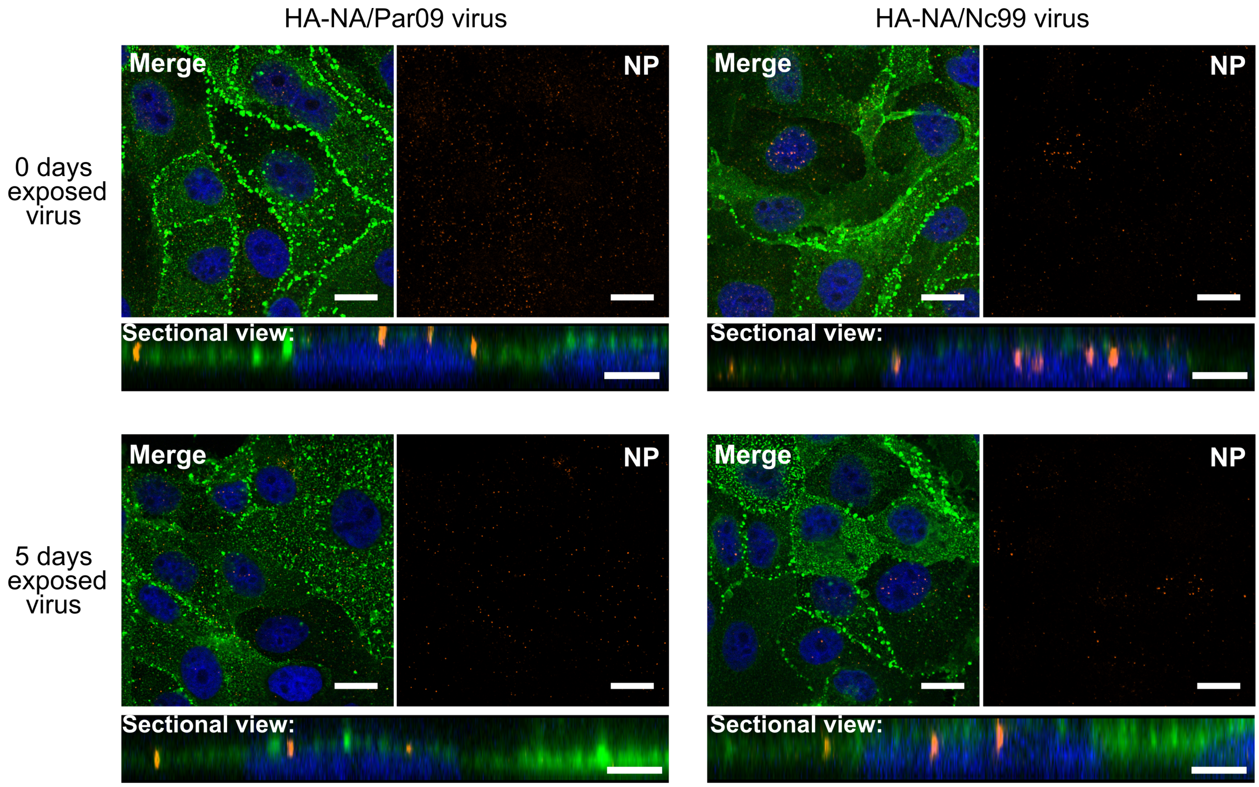

Supplementary Figure 2. Influenza NP immunolabelling 20 min after infection. Confocal immunofluorescence microscopy (x40) of MDCK cells 20 min after infection with either the HA-NA/Par09 virus (left) or the HA-NA/Nc99 virus (right) which were exposed (bottom) or not (top) for 5 days to saline water (35 $g.L^{-1}$ NaCl) at 35°C. Signal of the influenza NP immunolabelling is shown in orange. WGA labelling signal at the cell membrane is shown in green and the nucleus labelling with Hoescht dye is shown in blue. For each condition a merge of all channels (top left), the NP immunolabelling acquisition channel only (top right) or a Z-stack of sectional views at a distance of 0.42 µM (bottom) are shown. Scale bars represent 20 µm (merged and NP views) and 10 µm (sectional views).

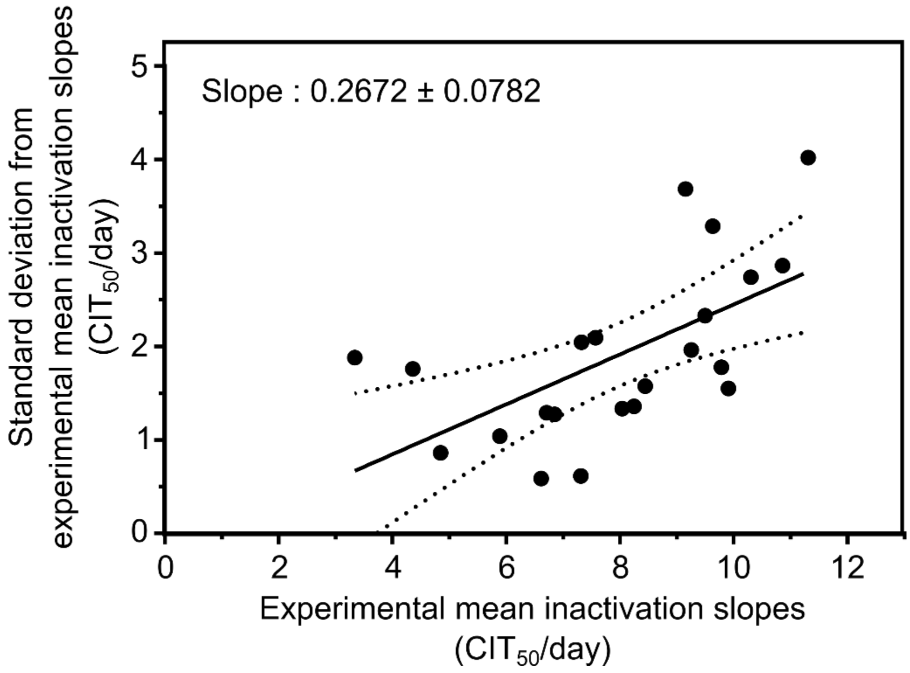

Supplementary Figure 3. Standard deviation values tend to increase when the mean inactivation slopes are higher. All standard deviations calculated with experimental mean inactivation slopes were analysed with a linear regression method (solid line). Dashed lines represent the 95% confidence intervals.

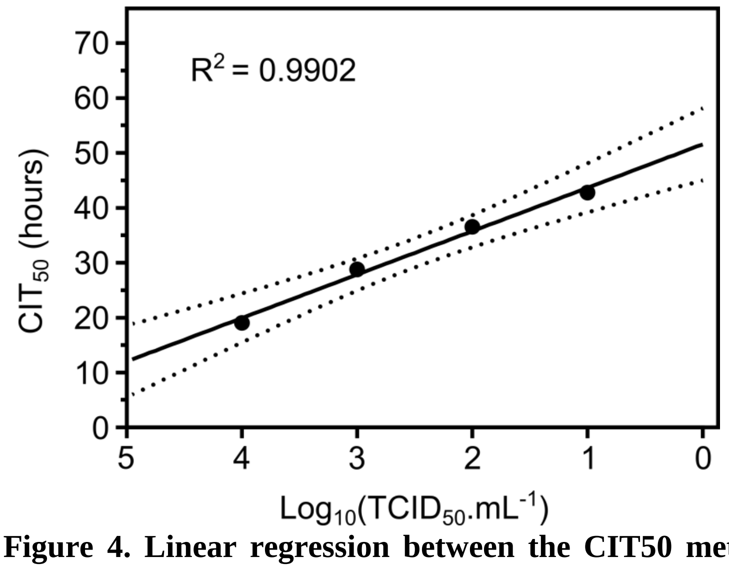

Supplementary Figure 4. Linear regression between the CIT50 method and the TCID50 method used for infectious particles quantification. CIT_{50} value (in hours) is linearly correlated to the number of infectious particles quantified by $TCID_{50}$ (wild-type A/NewCaledonia/20/1999 virus). Solid line represents the linear regression slope and dashed lines represent the 95% confidence intervals.
